# Extranodal Diffuse Large B-cell Lymphoma With Primary Clinical Presentation as Acute Cholecystitis: A Case Report

**DOI:** 10.7759/cureus.37552

**Published:** 2023-04-14

**Authors:** Fabian Rössler, Alexandra Sachs, Uwe Bieri, Boris Kuzmanic, Veronika Ballova, Ulrich Schneider, Gad Singer, Antonio Nocito

**Affiliations:** 1 Department of Surgery and Transplantation, Universitätsspital Zürich, Zürich, CHE; 2 Department of Internal Medicine, Spital Limmattal, Zürich, CHE; 3 Department of Urology, Kantonsspital Chur, Chur, CHE; 4 Department of Pathology, Kantonsspital Baden, Baden, CHE; 5 Department of Oncology, Kantonsspital Baden, Baden, CHE; 6 Department of General, Visceral and Vascular Surgery, Kantonsspital Baden, Baden, CHE

**Keywords:** acute cholecystitis, case report, non-hodgkin lymphomas, gallbladder, diffuse large b-cell lymphoma

## Abstract

This case describes a rare presentation of a diffuse large B-cell lymphoma not otherwise specified (DLBC NOS) in the gallbladder. We report the case of an 89-year-old male who initially presented with a two-week history of weakness and abdominal discomfort. We performed laparoscopic cholecystectomy for suspicion of acute cholecystitis. After the initial uneventful course, readmission occurred for persisting weakness a few weeks after surgery. Computed tomography revealed progressive retroperitoneal lymphadenopathy. With new emerging neurological symptoms, taking into account the histopathological findings of the gallbladder specimen, the diagnosis of DLBCL NOS was confirmed. Due to the rapid clinical deterioration and extranodal involvement, the patient opted against further therapy. When the suspicion of cholecystitis is inconclusive, rare differential diagnoses need to be considered. This analysis may improve the understanding of the presentation and course of DLBC NOS in abdominal organs and could form the basis for a systematic review to improve diagnosis and therapy.

## Introduction

Most gallbladder tumors are adenocarcinomas; however, around 0.1-0.2% are malignant lymphomas [1]. On the other hand, lymphomas occur in about 40% of the cases in extranodal tissue [1], but only in very few cases in the gallbladder [2]. There are only 68 cases of lymphoma manifestation in the gallbladder described in the English-language literature in PubMed from 1985 to 2020 and even fewer cases of diffuse large B-cell lymphoma not otherwise specified (DLBCL NOS) (n = 15) [2]. We report a rare case of a primary presentation of DLBCL NOS in the gallbladder due to typical histological findings of perivascular accumulation of malignant cells after laparoscopic cholecystectomy for suspected cholecystitis. The presented case is reported in line with the CARE guidelines.

## Case presentation

An 89-year-old male presented himself at the emergency ward with abdominal pain, a two-week history of general weakness, and a 3 kg weight loss. The patient’s chart revealed a known seropositive oculobulbar myasthenia gravis treated with Mestinon (pyridostigmine) and Imurek (azathioprine), a hypertensive, valvular, and rhythmogenic heart disease, a severe chronic renal failure, an amiodarone-induced thyroid insufficiency, a nonmuscle invasive bladder cancer, and presence of low-risk prostate cancer. He had a positive family history of cardiovascular disease as his father had known hypertension and his brother had suffered a stroke. Still, there was no history of malignant neoplasm in the family. Physical examination revealed abdominal tenderness with local peritonism in the left lower abdomen. Otherwise, the patient showed no fever and normal cardiopulmonary status. The patient’s C-reactive protein (CRP) was slightly elevated (14.6 mg/L, reference: <5 mg/L) while having a normal white blood cell count (WBC). Other laboratory tests were unremarkable apart from the known reduced renal function (with an estimated glomerular filtration rate of <30 mL/minute) and elevated cardiac parameters; however, possibly influenced by the impaired kidney function. Due to the known renal insufficiency, only a native computed tomography (CT) scan of the abdomen was obtained, showing suspected diverticulitis and cholecystolithiasis with equivocal radiographic signs of cholecystitis as well as retroperitoneal lymphadenopathy.

Due to the clinical and radiographic findings, antibiotic therapy with ceftriaxone and metronidazole was established to treat diverticulitis. Over the course of the following days, the laboratory parameters revealed a further increase in CRP and a decrease in WBC below 3 G/L (reference: 3.0-9.6 G/L), accompanied by clinical worsening of the patient despite the established empiric antibiotic treatment. Due to the reported findings and a new aversion to eating meat, abdominal sonography was conducted showing at this time typical signs of acute cholecystitis with suspicion of a small pericholecystitis abscess formation. The patient underwent laparoscopic cholecystectomy and the short-term course was uneventful. A few days after surgery, the patient was discharged from the hospital in a much-improved condition at his request due to urgent personal matters.

Two weeks later, the patient was readmitted to the hospital due to increasing weakness, inappetence, and recurring pulpy diarrhea. The CT scan on admission revealed ascites, pan-diverticulosis with diffuse accentuated intestinal mucosa, inhomogeneous liver parenchyma, and a progression of retroperitoneal lymphadenopathy. Furthermore, the patient now complained of a newly perceived numbness of the chin.

**Figure 1:**
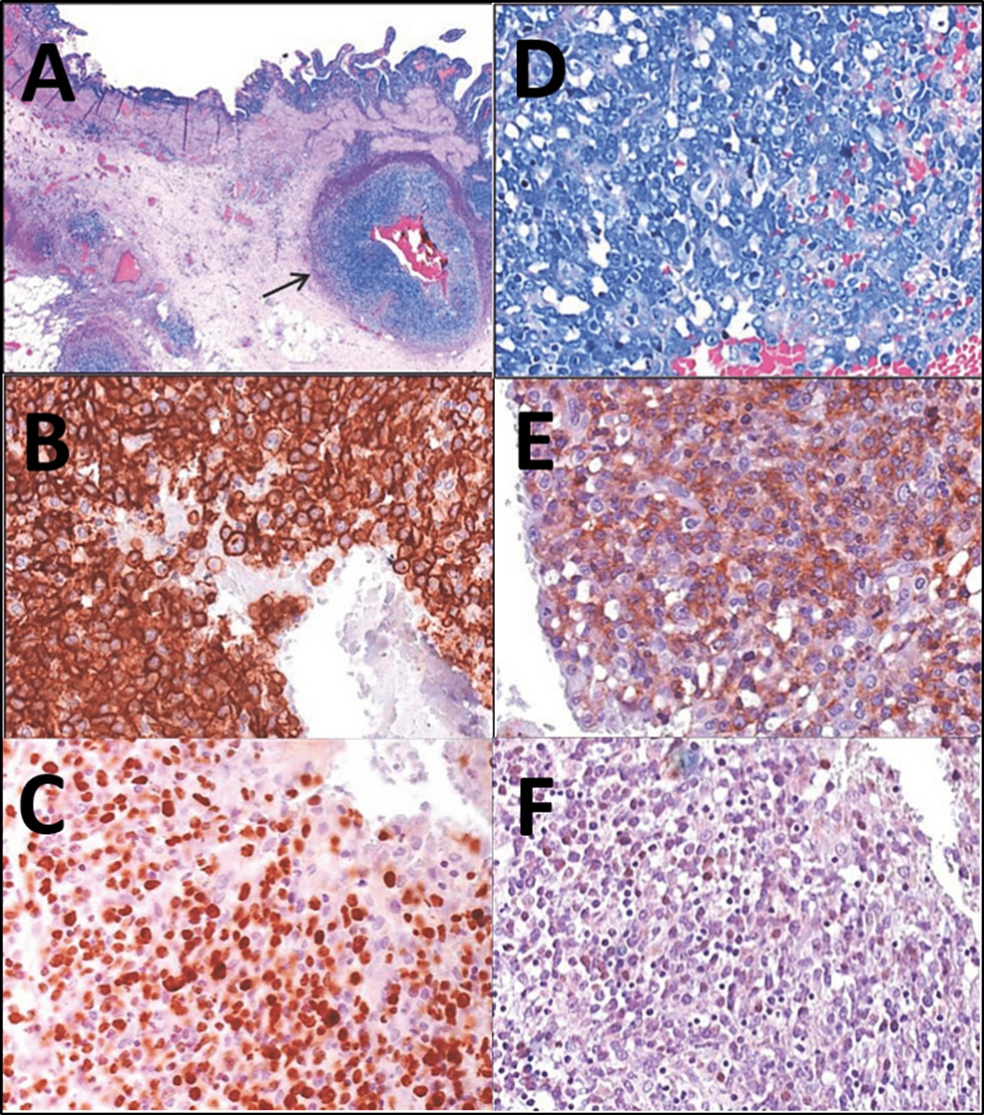
Histopathological stainings and immunohistochemical examinations. A: Overview (hematoxylin and eosin, ×25) of the dense perivascular arrangement of lymphocytes. B: Immunohistochemical positivity of blasts for CD20 (400×). C: Ki67 shows a high proliferation rate of over 70% (400×). D: Infiltrate of atypical blasts with prominent nucleoles (hematoxylin and eosin, 400×). E: Immunohistochemical expression of CD5 (400×). F: Negative in situ hybridization for Ebstein-Barr virus RNA (400×).

The definitive histopathological findings of the cholecystectomy specimen showed DLBCL NOS, non-germinal center B-cell (GCB)-type. Among chronic inflammation of the gallbladder wall, it showed a striking perivascular infiltrate of large, atypical lymphocytes (Figure 1A). The immunohistochemical subtyping was characterized by CD20 positivity (Figure 1B) and a high proliferation rate of over 70% (Ki67 marker) (Figure 1C). Furthermore, infiltrates of atypical blasts with prominent nucleoles (Figure 1D) and coexpression of CD5 (Figure 1E) were present. The in situ hybridization for Epstein-Barr virus (EBV) RNA was negative (Figure 1F). The negative in situ hybridization for EBV RNA and the absence of microvascular clots excluded the possible differential diagnosis of fibrin-associated large B-cell lymphoma as well as lymphomatoid granulomatosis [3,4]. The absence of intravascular lymphoid elements and the predominant perivascular localization contradicted the differential diagnosis of an intravascular large B-cell lymphoma, despite the immunohistochemical expression of CD20 and CD5 [4,5]. In this case, the rare finding of CD5 expression was associated with poor prognosis [6-9].

**Figure 2:**
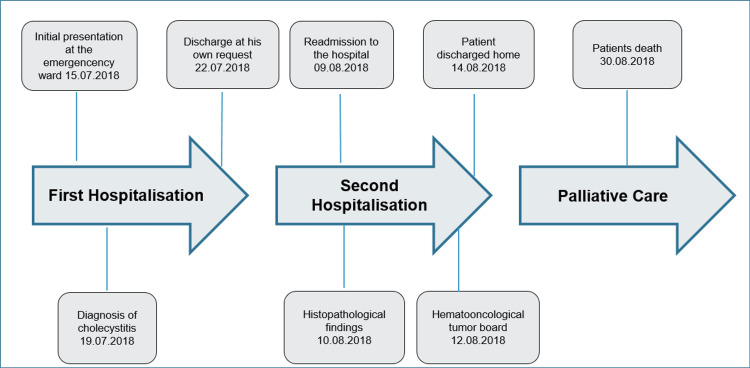
Course of the disease from initial admission to death, emphasizing critical periods in the diagnostic and therapeutic process.

After discussing the case on the hemato-oncological tumor board, completion of staging with a positron emission tomography (PET) scan, a spinal tap, and a colonoscopy was suggested along with the induction of chemotherapy with R-bendamustine instead of R-CHOP (rituximab, cyclophosphamide, doxorubicin, vincristine, prednisone) due to the comorbidities of the patient. After explaining the need for further diagnostics and the therapeutic options to the patient, he declined all further measures and was discharged home with palliative care nursing services. The complete course is described in Figure 2.

## Discussion

We report a rare case of gallbladder lymphoma in an elderly patient with unspecific abdominal symptoms and initial suspicion of acute cholecystitis. The patient deteriorated a few weeks after laparoscopic cholecystectomy and developed newly emerged neurological symptoms, while the CT scan showed progressive retroperitoneal lymphadenopathy. The histopathological finding of the cholecystectomy specimen corresponded with the diagnosis of DLBCL NOS, a neoplasm usually divided into two molecular subgroups, namely, the GCB alike and activated B-cell-alike (ABC) or non-GCB-type according to their gene expression profile (GEP). For the non-GCB type, the prognosis is generally poor [10]. Because GEP is not generally available, various immunohistochemical algorithms have been developed, none of which are specifically recommended by the World Health Organization (WHO). The 2016 revision of the WHO classification of aggressive B-cell lymphomas emphasizes immunohistochemical coexpression of MYC and BCL2 proteins as markers for poor prognosis. Such double-expressor lymphomas are associated with adverse outcomes when treated with R-CHOP [10]. Astonishingly, most of them are GCB type, which normally has a better prognosis than ABC types with R-CHOP treatment. Furthermore, the composition of the microenvironment and genetic aberrations of antigen-presenting functions and immune recognition will probably increase therapeutic options in the future [10].

DLBCL accounts for approximately 30% of non-Hodgkin lymphomas in adults [10]. Compared to other case reports of DLBCL of the gallbladder, our patient was relatively old (89 years vs. 60.9 years on average) [2]. Symptoms at initial admission, however, mimic acute cholecystitis in more than 90% of cases [11]. In this case, however, the patient did not develop a fever during the disease course. Definitive diagnosis is often delayed and eventually ensured by the histopathological workup [2]. Furthermore, 10 of the 15 published cases were about male patients [2]. The clinical presentation with a primary extranodal manifestation in the gastrointestinal tract, i.e., the gallbladder, and secondary neurological clinical features, i.e., numbness of the chin, which could not be histologically or radiographically confirmed, correspond with the diagnosis of a DLBCL NOS. We could not find a similar case in the existing literature, although there are a few intravascular large B-cell lymphoma mimicking acute cholecystitis described [12-15].

## Conclusions

Despite incomplete staging, this case report can contribute to developing a better understanding of this rare entity. Future research is needed as, for example, an autopsy-based case series of DLBCL NOS patients investigating the visceral organs systematically for perivascular lymphocyte accumulation. However, treatment options are a topic of ongoing research due to the heterogeneous nature of DLBCL NOS. Investigation of MYC and BCL2 status can improve the estimation of individual prognosis.
